# First interim analysis of a randomised trial of whole brain radiotherapy in melanoma brain metastases confirms high data quality

**DOI:** 10.1186/s13104-015-1153-5

**Published:** 2015-05-08

**Authors:** Gerald B Fogarty, Angela Hong, Kari Dolven-Jacobsen, Claudius H Reisse, Bryan Burmeister, Lauren H Haydu, Haryana Dhillon, Victoria Steel, Brindha Shivalingam, Kate Drummond, Janette Vardy, Anna Nowak, George Hruby, Richard A Scolyer, Catherine Mandel, John F Thompson

**Affiliations:** Melanoma Institute Australia, Sydney, Australia; Department of Radiation Oncology, St Vincent’s General Hospital, Sydney, Australia; Genesis Cancer Care, Department of Radiation Oncology, Mater Hospital, Sydney, Australia; Trans-Tasman Radiation Oncology Group (TROG), Newcastle, Australia; Sydney Medical School, |The University of Sydney, Sydney, Australia; Royal Prince Alfred Hospital, Sydney, Australia; Oslo University Hospital HF, The Norwegian Radium Hospital, Oslo, Norway; Princess Alexandra Hospital, Brisbane, Australia; Centre for Medical Psychology & Evidence-based Decision-making, Sydney Medical School, The University of Sydney, Camperdown, NSW Australia; Psycho-Oncology Co-Operative Research Group (PoCoG), School of Psychology, Faculty of Science, University of Sydney, Sydney, Australia; Australia and New Zealand Melanoma Trials Group (ANZMTG), North Sydney, Australia; The Royal Melbourne Hospital & University of Melbourne, Parkville, Australia; School of Medicine and Pharmacology, University of Western Australia, Crawley, Australia; Department of Medical Oncology, Sir Charles Gairdner Hospital, Nedlands, Australia; Peter MacCallum Cancer Centre East Melbourne & University of Melbourne, Parkville, Australia; Concord Repatriation and General Hospital, Concord, Australia

**Keywords:** Radiotherapy, Metastases, Melanoma, Brain, Whole brain radiotherapy, Randomised trial

## Abstract

**Background:**

Brain metastases are a common cause of death in patients with melanoma. The role of adjuvant whole brain radiotherapy (WBRT) following local treatment of intracranial melanoma metastases is controversial. The Australian and New Zealand Melanoma Trials Group (ANZMTG) and the Trans-Tasman Radiation Oncology Group (TROG) are leading the first ever single histology randomised trial investigating this question. The primary endpoint is distant intracranial failure on magnetic resonance imaging (MRI) within twelve months of randomisation. The first planned interim analysis was performed twelve months after randomisation of the 100^th^ patient. The analysis was an opportunity to review completeness of the trial data to date.

**Methods:**

All data received up to the end of twelve months after randomisation of the 100th patient was reviewed.

**Results:**

Review of pathology reports confirmed that all 100 patients had stage IV melanoma and were appropriately entered into the study. Of the 47 distant intracranial events, 34 occurred in isolation (i.e. only distant failure was identified), whilst 13 were accompanied by local failure. Data review showed compliance with the protocol mandated MRI schedule and accuracy of intracranial failure reporting was very high. The Quality of Life (QoL) component of the study achieved a 91% completion rate. For the neurocognitive function (NCF) assessments, a high completion rate was maintained throughout the 12 month period. Where assessments were not performed at expected time points, valid reasons were noted. Radiotherapy quality was high. Of 50 patients who received WBRT, 32 were reviewed as per protocol design and there was only one major variation out of 308 data points reviewed (0.3%). There were minimal trial related adverse events (AEs) and no serious adverse events (SAEs). Pre-specified protocol stopping rules were not met.

**Conclusions:**

The Data Safety Monitoring Committee (DSMC) recommended the trial continue recruitment after reviewing the unblinded data. The data provision and quality to date indicates that a reliable outcome will be obtained when the final analysis is performed. Accrual is ongoing with 156 out of 200 patients randomised to date (26^th^ November 2014).

## Background

Brain metastases (BMs) are common in patients with metastatic melanoma [1,2] and are the cause of death in the majority of them [2,3]. Whole brain radiotherapy (WBRT) is a common adjuvant treatment following local treatment of BMs with neurosurgery and/or stereotactic irradiation (SRS). However, there is no specific level one evidence to support this approach in melanoma. Proponents believe that WBRT improves palliation by prolonging intracerebral control [4]. Critics state that WBRT does not increase survival, may cause neurocognitive problems and may not prevent intracerebral progression [5,6]. A randomised controlled trial (RCT) is needed to resolve this controversy in melanoma.

The Australia and New Zealand Melanoma Trials Group (ANZMTG) and Trans-Tasman Radiation Oncology Group (TROG) are leading a RCT investigating this question. This trial is known as the **w**hole **b**rain **r**adio**t**herapy in **mel**anoma (WBRTMel) trial. The trial protocol has previously been published [7]. The primary endpoint is distant intracranial failure on magnetic resonance imaging (MRI) within twelve months of randomisation. The total number of patients required was calculated as being 200, and a feasibility study was completed and reported [8].

A planned interim analysis performed at twelve months after randomisation of the 100^th^ patient was triggered in December 2013. Clinicians remain blinded to the study results. The interim analysis presented an opportunity to review completeness of the trial data to date and these data are now presented.

## Methods

**Figure 1:**
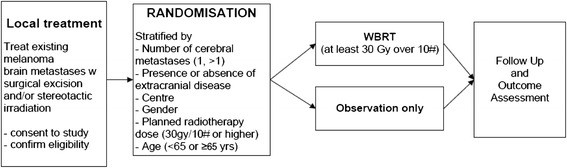
Trial Schema.

This is an international, multi-centre, open-label, stratified, 2-arm randomised phase III trial (Figure 1).

The trial has been approved by the Cancer Institute NSW Clinical Research Ethics Committee #2007C/11/032 and relevant hospital ethics committees in each participating centre. Written informed consent for participation in the study is obtained from all participants.

The first recruiting centre was opened in December 2008, with the first trial patient randomised in April 2009. The 100^th^ patient was randomised on 3^rd^ December 2012, triggering the first planned interim analysis in December 2013. Data collected until that date was reviewed.

Data were reviewed for quality, and the primary endpoint analysed. Histopathology reports (of primary tumour or metastases) of all 100 patients were independently reviewed. Data recorded on MRI Case Report Forms (CRFs) were compared with corresponding MRI reports. Quality of life (QoL) and neurocognitive function (NCF) data were reviewed for completeness. Radiotherapy (RT) quality review was conducted by TROG Quality Assurance (QA) Team. The first 5 patients from each site randomised to receive whole brain radiotherapy were reviewed for RT QA. Subsequently, patient reviews followed a 1 in 5 random sampling basis from each site, in accordance with the standard TROG QA protocol for all RT trials. A random sample of 10% of the first 100 patients had all critical data points reviewed for accuracy of entry. Safety data for all 130 patients entered onto the trial at the time of the analysis were reviewed.

Whilst trial results remain blinded to clinicians and the Trial Management Committee (TMC), the independent Data Safety Monitoring Committee (DSMC) were presented with the unblinded results (i.e. when reviewing the primary endpoint of the trial, they could ascertain which arm of the trial each patient had been assigned to). The DSMC were also provided with the most recent safety data for all trial patients. The recommendations of the DSMC were presented to the TMC to decide whether to continue the trial.

## Results

### Demographics

Demographic information is presented (Table 1). Briefly, baseline analysis demonstrated even distribution between the treatment groups (50 patients per arm). The 100 patient cohort included 70% males and 30% females, aged between 26 and 83 years. Mean ranges of Australian patients were marginally older than international patients entering the trial (61 years vs 58 years), but balanced between treatment arms (WBRT 61 years vs Observation 59 years). Fifty-nine percent of the first 100 patients presented with a solitary brain metastasis, and the remainder with two (29%) or three metastases (12%). Twenty-seven percent of patients presented at baseline with no evidence of extracranial disease. Demographics reported in this analysis of the first 100 patients are consistent with the results of our feasibility study [8].

**Table 1:** **Patient demographics (First 100 patients)**

**Site**	**No. of Patients**
Treatment Centre	
Australian	68
International	32
Gender	
Male	70
Female	30
Mean Age (years)	Mean 61 (Australian) Mean 58 (International) Range: 26 to 83
Local treatment of BMs	
Neurosurgery	62
Stereotactic (Radiosurgery (SRS)	24
Combination of Neurosurgery and SRS	13
None*	1
Number of Brain Mets	
1	59
2	29
3	12
Extracranial Disease	
Present	73
Absent	27

*One patient died prior to receiving local treatment to their brain metastases.

BM – brain metastases.

### Primary endpoint

**Figure 2:**
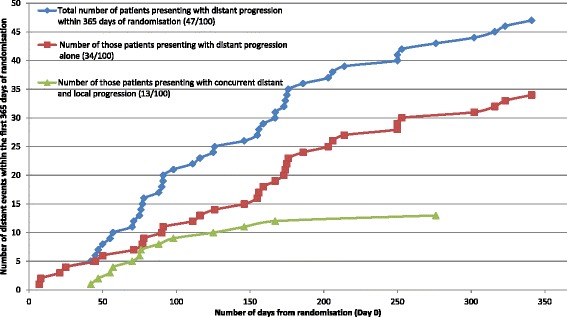
Number of patients presenting with distant intracranial progression within 365 days of randomisation for the first 100 patients accrued to the ANZMTG 01.07 WBRTMel Trial.

The primary endpoint was distant intracranial failure on MRI within one year. The primary endpoint was reached in 47 of the first 100 patients: 34 experienced distant intracranial failure only, while in 13 patients it was accompanied by local failure (Figure 2).

### Protocol compliance

MRI schedule data review revealed high protocol compliance; with 81 of the 100 patients having MRI scans performed as per protocol (i.e. every 3 months until distant intracranial progression was reported or until death). Seven patients did not have one or more of their scheduled MRI scans within the first 12 months. One patient had distant failure detected on computed tomography (CT) scan. Twelve patients had further MRI scans, despite confirmation of distant failure. The accuracy of distant intracranial failure reporting was 100%, but stringent quality assurance checks highlighted some minor inaccuracies requiring correction despite their lack of impact on critical end points. Examples of these are outlined below:

- Only distant failure was reported, when a local failure was present and should also have been documented (n = 1).
- Information detailed in the MRI report was found to be wrong and the CRF was correct (n = 1).
- A local failure was reported twice, as the site had reported continued growth of a lesion that had already been recorded as a failure (n = 1).
- For one instance of failure, only one distant lesion was recorded when two had been identified with the MRI report (n = 1).

Of 50 patients who received WBRT, 32 were reviewed (Table 2). There was only one major variation among the 308 data points reviewed (0.3%). This was due to a patient whose RT delivery time extended beyond 10% of that expected due to a treatment break for concomitant illness. Treatment was subsequently completed.

**Table 2:** **Radiotherapy quality assurance data for 32 patients in the interim analysis**

**Categories**	**Variables reviewed**	**Acceptable**	**Minor variation**	**Major variation**	**Missing**
**Schedule**	84	82	1	1	0
**Dose**	110	110	0	0	0
**Technique**	58	56	2	0	0
**Documentation**	56	56	0	0	0
**Total**	**308**	**304(98.7%)**	**3(1.0%)**	**1(0.3%)**	**0**

### Data quality and safety

Data submitted from all sites were reviewed centrally for completion. Pathology report review confirmed all 100 patients had evidence of AJCC stage IV melanoma and were appropriately entered into the study. Completion of the QoL questionnaires (EORTC QLQC30 and BN20) has been excellent, with a 91% completion rate. For those patients participating in the two monthly NCF assessments (those recruited from Norway were excluded from this component of the trial as English fluency to grade 8 level was a prerequisite), compliance has been high. At baseline 99% of expected assessments were completed. This completion rate decreased overtime (Table 3), however stands at 59% at 12 months. When assessments were not performed at expected time points, valid reasons were noted (e.g. patient too unwell).

**Table 3:** **NCF assessment completion**

**Time point**	**Baseline**	**Month 2**	**Month 4**	**Month 6**	**Month 8**	**Month 10**	**Month 12**
**Proportion of expected NCF assessments completed (%)**	99	91	78	68	67	52	59

For each time point the number of NCF assessments expected to be completed was determined and compared with the number of NCF assessments actually completed. From this the proportion of expected NCF assessments completed were calculated and is displayed in Table 3.

For 10 randomly selected patients, critical data points within the database were reviewed including the Eastern Cooperative Oncology Group (ECOG) Performance Status scores, the NCF and QoL data, leading to verification of 4,440 data fields. From this, a minimal data entry error rate of 0.2% was determined. Safety data review showed that adverse events (AEs) were minimal, with only 47 events of grade 3 or 4 occurring (Table 4). No serious adverse events (SAEs) have been reported to date. None of the protocol specified stopping rules were met.

**Table 4:** Adverse events*

**Adverse event**	**No. of Grade 3 events**	**No. of Grade 4 events**
Anorexia	5	0
Aphasia	1	0
Cellulitis	1	0
Dehydration	1	0
Disseminated Intravascular Coagulation	0	1
Fatigue	15	2
Gait/Walking disturbance	2	0
Hemiparesis	1	0
Hypokalaemia	1	0
Hyponatraemia	1	0
Muscle weakness	2	0
Nausea	1	0
Pain	3	0
Peripheral motor neuropathy	1	0
Reduced sight	1	0
Renal impairment	1	0
Seizure	2	0
Thromboembolitic event	1	0
Vomiting	3	0
Weight loss	1	0
**Total number of AEs at specified grade =**	**44**	**3**
**Total number of AEs =**	**47**	

*All AEs have been classified in accordance with CTCAE v 4.0.

## Discussion

The DSMC reviewed the unblinded primary end point interim analysis data and the corresponding safety data for this trial. They stated that the trial is progressing in a manner that is safe for patients, the data collected are of an excellent standard, and no protocol defined stopping rules have been met. Therefore they recommended the trial continue to completion. Based on this recommendation, the TMC, blinded to the primary end point analysis, approved the continuation of the trial. Recruitment to the trial continues with 156 out of 200 patients currently randomised (26^th^ November 2014). The primary end point interim analysis data remains blinded to the WBRTMel Operations Team, the TMC and all investigators, clinicians, site staff, and patients.

For the primary endpoint of distant intracranial progression at 12 months, of the total cohort there were 47 distant intracranial events; 34 occurred in isolation (i.e. only distant failure was identified), whilst 13 were accompanied by local failure. This finding indicates that the trial selection criteria are appropriately identifying a population at high risk of distant intracranial failure, and this risk is similar to that reported in other studies. In a recent retrospective review, Dyer *et al*. [9] reported 86 of 147 (59%) patients developed distant intracranial progression at a median of 4.3 months. The addition of adjuvant WBRT on multivariate analysis being associated with significantly prolonged intracranial control (HR 2.24, p = 0.005), especially for those with more than one BM.

Protocol compliance and high data quality are essential for ensuring that the trial results are reliable. These results compare favourably with other practice-changing clinical trials [10,11]. With regards to the NCF component the WBRTMel trial will provide a more comprehensive data set than previous trials [12]. The introduction of hippocampal avoidance to the protocol, at centres able to give WBRT in this manner, will also provide an additional perspective on WBRT. At the point of final analysis the NCF of patients who received observation vs WBRT with hippocampal avoidance vs WBRT without hippocampal avoidance will be reviewed. The interim analysis has allowed an opportunity to recheck and clean the data to eradicate errors, both systematic and non-systematic, before the final analysis, due twelve months after the last patient is randomised. Quality is especially important in RT trials, some of which may have foundered because of lack of RT quality control [13].

## Conclusion

RCTs are fundamental to improving clinical care via the use of evidence–based medicine. Based on a planned interim analysis the WBRTMel trial is safe and with data of high quality. The trial is on track to answer an important controversy in melanoma management and as far as we are aware, remains the first ever reported single tumour type WBRT trial. The full trial continues, with 156 patients of the target 200 randomised to date (26^th^ November 2014).

## Abbreviations

AEs: Adverse events
ANZMTG: Australia and New Zealand Melanoma Trials Group
BMs: Brain metastases
CRFs: Case report forms
CT: Computed tomography
DSMC: Data Safety Monitoring Committee
ECOG: Eastern Cooperative Oncology Group
EORTC: QLQ BN20 European Organisation for Research and Treatment of Cancer Quality of Life Questionnaire (brain cancer specific)
EORTC: QLQ C30 European Organisation for Research and Treatment of Cancer Quality of Life Questionnaire (cancer specific)
HR: Hazard ratio
ICHGCP: International Congress of Harmonization and Good Clinical Practice
MRI: Magnetic resonance imaging
NCF: Neurocognitive function
OBS: Observation
QA: Quality assurance
QoL: Quality of life
RCT: Randomised controlled trial
RT: Radiotherapy
SAEs: Serious adverse events
SRS: Stereotactic irradiation
TMC Trial: Management Committee
TROG: Trans-Tasman Radiation Oncology Group
Tx: Treatment
WBRT: Whole brain radiotherapy
WBRTMel Whole: brain radiotherapy in melanoma – acronym of this trial
